# A journey with type IX secretion system effectors: selection, transport, processing and activities

**DOI:** 10.1099/mic.0.001320

**Published:** 2023-04-12

**Authors:** Maëlle Paillat, Ignacio Lunar Silva, Eric Cascales, Thierry Doan

**Affiliations:** ^1^​ Laboratoire d'Ingénierie des Systèmes Macromoléculaires (LISM), Institut de Microbiologie, Bioénergies et Biotechnologie (IM2B), Aix-Marseille Université – CNRS, UMR 7255, Marseille, France

**Keywords:** protein transport, secretion signal, effectors, toxins, type IX secretion, gliding motility

## Abstract

The type IX secretion system (T9SS) is a multiprotein machine distributed in *Bacteroidota* and responsible for the secretion of various proteins across the outer membrane. Secreted effectors can be either delivered into the medium or anchored to the cell surface. The T9SS is composed of a transenvelope complex consisting of proton-motive force-dependent motors connected to a membrane-associated ring and outer membrane translocons, and a cell-surface anchoring complex that processes effectors once translocated. The T9SS is involved in pathogenesis, metal acquisition, carbohydrate degradation, S-layer biogenesis and gliding motility. The broad spectrum of functions is linked to a highly versatile repertoire of effectors including metallophores, enzymes, toxins and adhesins, that all possess specific signatures to be recruited and transported by the apparatus. This review summarizes the current knowledge on T9SS substrate secretion signals, transport, processing and activities.

## Introduction

The type IX secretion system (T9SS) is a modular protein transport machinery that is assembled into the cell envelope of Gram-negative bacteria of the phylum *Bacteroidota *, such as members of the genera *Flavobacterium *, *Porphyromonas *, *Cytophaga *, *Cellulophaga *, *Riemerella* and *Capnocytophaga *, with the exception of *Bacteroides* species [1–5]. The broad but strict distribution of T9SS genes within the phylum and the observation that T9SS conserved genes are scattered within the genomes suggest an ancient acquisition, probably in a common ancestor of *Bacteroidota* [6]. In these different species, the T9SS is used to secrete a very large repertoire of effectors or enzymes across the outer membrane in order to facilitate or sustain the lifestyle of the bacterium [6–10]. The T9SS is therefore involved in many processes such as virulence, S-layer formation, gliding motility, and biopolymer degradation and utilization [2, 11, 12]. Due to the use of these different processes as read-out to identify T9SS components, homologous genes have received distinct names in different bacterial species. Awaiting a unified nomenclature, we will use in this review the *Porphyromonas* and *Flavobacteria* names, which are the most widely used in the literature: Por for *Porphyromonas* secretion, Gld for gliding or Spr for spreading.

## T9SS architecture and mechanism of action

**Fig. 1.**
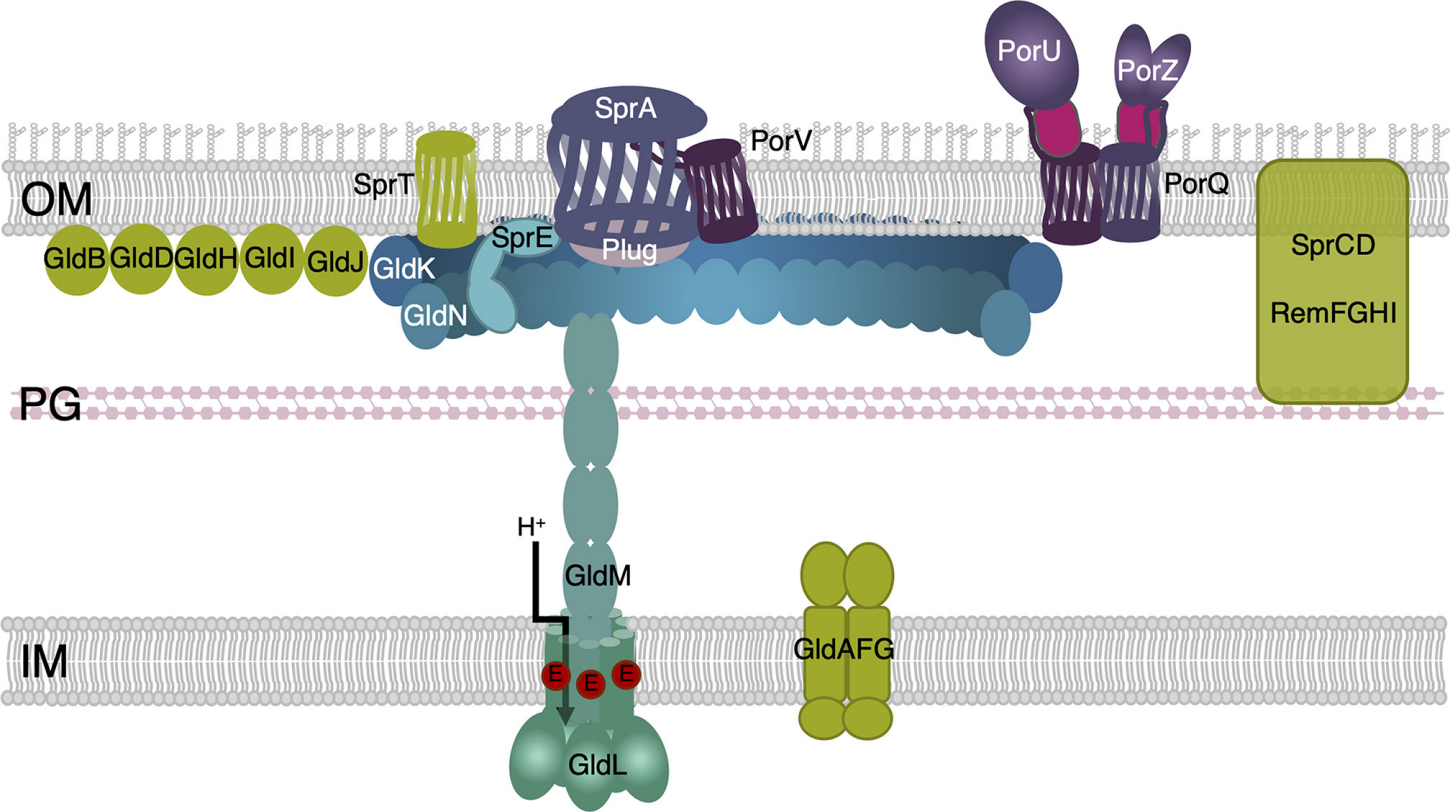
Schematic representation of the type IX secretion system (T9SS) and of associated proteins and modules. The major subunits of the T9SS are schematized (shade of blue), highlighting the LM rotary motor, KN ring, the SprE/PorW lipoprotein and the Sov/SprA translocon associated with Plug (grey). For the rotary motor, the glutamate residue that is thought to harvest the proton (H^+^) gradient during motor function is shown in red. The PorV β-barrel and the PorQUZ attachment complex are shown in purple. Additional modules associated with type IX secretion or gliding motility are depicted in green. IM, inner membrane; PG, peptidoglycan; OM, outer membrane.

The core subunits of the T9SS can be categorized into three subcomplexes: the PorLM/GldLM rotary motor, the PorKN/GldKN outer membrane-associated structure and the Sov/SprA translocon (Fig. 1) [1, 4, 5]. A number of additional subunits could be associated with these subcomplexes such as GldO and GldJ associated with the GldKN complex in *Flavobacterium *, and the PorV outer membrane β-barrel and Plug proteins that bind the Sov/SprA translocon [5, 6].

### The rotary motor

The PorL/GldL and PorM/GldM inner membrane proteins assemble an L:M complex of 5 : 2 stoichiometry [13–15]. The PorL/GldL protein comprises two transmembrane helices (TMHs) and a cytoplasmic domain, whereas the PorM/GldM protein is a bitopic protein with a single N-terminal TMH of in-to-out topology followed by a large periplasmic domain complex [13]. The structures of the PorM/GldM periplasmic regions have been resolved: the two monomers are tightly associated and are organized in a four-domain stick shape, with a helical D1 domain and three Ig-like D2–D4 domains which present swapping β-strands between dimers in domains D2 and D3 [16, 17]. In the inner membrane, the ten helices of the PorL/GldL pentamer wrap the two helices of PorM/GldM (Fig. 1) [14, 15]. The structures of the inner membrane-associated portion of the LM complexes from various *Bacteroidota* T9SS have been reported, revealing a composition, organization and structure homologous to proton-motive force (PMF)-driven motors such as MotAB, ExbBD, TolQR and AglQRS, involved in energizing flagellum rotation, TonB-dependent iron and nutrient uptake, cell envelope stability or *Myxococcus* gliding motility, respectively [14, 15]. Indeed, *Flavobacterium* gliding motility is energized by the PMF, and more specifically by the proton gradient [8, 14, 18, 19]. Similarly to the other bacterial PMF-dependent molecular motors, a conserved acidic residue located in the PorL/GldL second TMH region is critical for function [14, 19]. It is thus proposed that the T9SS LM complex harvests and transduces the energy of the inner membrane proton gradient to drive protein transport through the T9SS [14, 19]. While the mechanism is not fully understood, it probably involves PMF-responsive conformational changes in the PorM/GldM periplasmic domain that may have downstream repercussions on the outer membrane (OM)-associated complex [19, 20].

### The OM-associated complex

The OM-associated complex is minimally composed of the PorK/GldK and PorN/GldN proteins [7, 13, 21]. In some species such as *Flavobacterium johnsoniae *, two additional subunits, GldJ (paralogue of GldK) and GldO (paralogue of GldN), also participate in complex formation [22–24]. PorK/GldK is an OM lipoprotein whereas PorN/GldN locates in the periplasm [13, 21]. In *Porphyromonas *, the PorK and PorN proteins interact and assemble a 36-mer, 50 nm large ring [20, 21]. This ring is located underneath the OM and is connected to 18 PorLM complexes via interactions between PorN and the PorM D4 domains, building an overall birdcage-like structure in the periplasm [13, 16, 20, 25]. It has been proposed that PorM conformational changes in response to the PMF induce the rotation of the PorKN ring [19, 20].

### The translocon

The translocon comprises a very large 36-strand β-barrel protein, Sov/SprA [26]. The barrel is closed at the extracellular side by exposed folded inserts and loops connecting the β-strands [26]. *Flavobacterium* SprA has been purified and imaged associated with the PorV β-barrel or to the Plug protein, probably reflecting two different states of the translocon activity [26]. The Plug protein associates and obstructs the periplasmic entrance of the translocon. In this conformation, the translocon presents a lateral aperture, which is penetrated by loops of the PorV β-barrel in the second conformation [26]. The current model proposes that alternating Plug and PorV association and dissociation may regulate effector transit through the translocon [26–28]. Cryo-tomography imaging suggested that eight translocons are confined within the OM-associated ring PorKN ring [20] and are probably connected to it by PorW/SprE lipoproteins [29].

### Additional modules

In addition to the core components described above, the T9SS can associate with other modules that confer specific functions (Fig. 1). In many species, a cell surface attachment module responsible for effector processing consists of the PorV and PorQ β-barrels, the PorZ protein that provides anionic lipopolysaccharide (A-LPS) to the PorU peptidase, which cleaves the C-terminal domain of effectors and eventually attaches them to the A-LPS [27, 30–35]. Other components and modules, such as the GldBDHI proteins and the ABC transporter-like GldAFG module, have no clear function but appear critical for gliding motility and indirectly for secretion through GldK stabilization [22, 24, 36–39]. The PorP/SprF proteins are PorV-like β-barrels, and can be deployed for the transport of specific substrates, notably for gliding adhesins. Finally, SprCD and Rem proteins are associated with gliding motility in *Flavobacteria* [40–42].

### T9SS: a two-step secretion mechanism

**Fig. 2.**
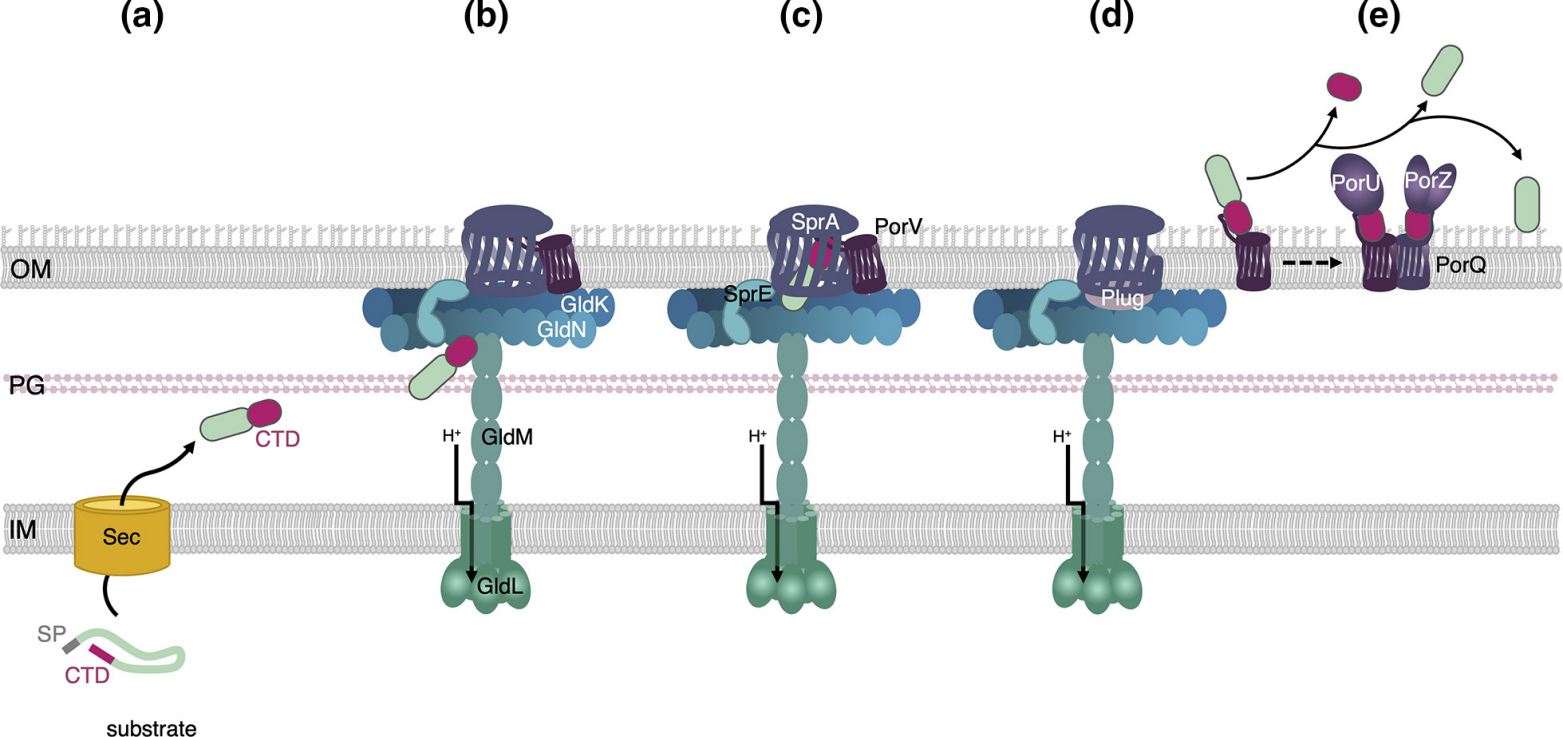
Schematic representation of the T9SS substrate transport pathway. The substrate, which possesses a signal peptide (SP), is exported to the periplasm via the Sec machinery (**a**). Once in the periplasm, the substrate folds, revealing a C-terminal domain (CTD, pink) that is recognized by the T9SS (**b**). The substrate is conveyed to the Sov/SprA translocon in the open conformation (**c**) where it is transferred to the PorV shuttle (**d**) and transported to the processing/attachment PorQUZ complex. The CTD is then cleaved by the PorU sortase and the protein is either released in the medium or anchored to the cell surface (**e**).

The overall mechanism of effector transport by the T9SS is not well understood but a model has been proposed based on recent findings on the apparatus. Type IX secretion is a two-step mechanism: all T9SS effectors possess an N-terminal Sec-dependent signal peptide and are thus exported from the cytoplasm to the periplasm by the Sec translocon in an unfolded conformation (Fig. 2a). It is proposed that the effectors fold in the periplasm prior to their recruitment to the T9SS. In the case of effectors with proteolytic activities, such as the *Porphyromonas gingivalis* gingipains, they fold as inactive zymogens in the periplasm, in which an N-terminal prodomain inhibits the enzymatic activity [43–45]. The specific recognition and selection of T9SS effectors is ensured by the presence of a C-terminal domain (CTD or T9SS-CTD) that serves as secretion signal [46]. While it is not known how the effectors engage the T9SS, they may bind to the OM-associated ring (Fig. 2b). The PorM/GldM conformational changes in response to PMF could induce rotation of the KN ring and thus distribute the effectors to the Sov/SprA translocons (Fig. 2c). Once inside the translocon, they may interact with the penetrating loops of the associated PorV β-barrel, which is thought to extract and shuttle them to the PorQUZ attachment complex. With PorV gone, the translocon periplasmic entrance is obstructed by the Plug protein preventing non-specific periplasmic leakage (Fig. 2d). The secreted effectors are processed by PorU and either released into the medium or attached to the cell surface (Fig. 2e).

## T9SS effector recognition and selection: the CTD secretion signal

As mentioned above, substrates of the T9SS are recognized and recruited to the apparatus in the periplasm via a conserved C-terminal domain, the CTD. The T9SS-CTD is necessary and sufficient for secretion, as its fusion to heterologous proteins promotes their secretion in a T9SS-dependent manner [47–49].

### Conserved sequence signatures

CTD-containing proteins are widespread among but restricted to members of the phylum *Bacteroidota *. Moreover, with few exceptions, the distribution of proteins with T9SS-CTDs strictly correlates with the distribution of T9SS core components [48]. CTDs can be categorized into two distinct protein domain families: type A and type B CTDs. Analysis of 104 *Bacteroidota* genomes identified >3000 proteins with type A CTD and about 900 proteins with type B CTD [48]. In addition, some proteins secreted by the T9SS such as the ChiA chitinase have a T9SS-CTD recently classified as type C CTD [50], which differs in sequence from the type A or the type B families [48].

### Type A CTD

**Fig. 3.**
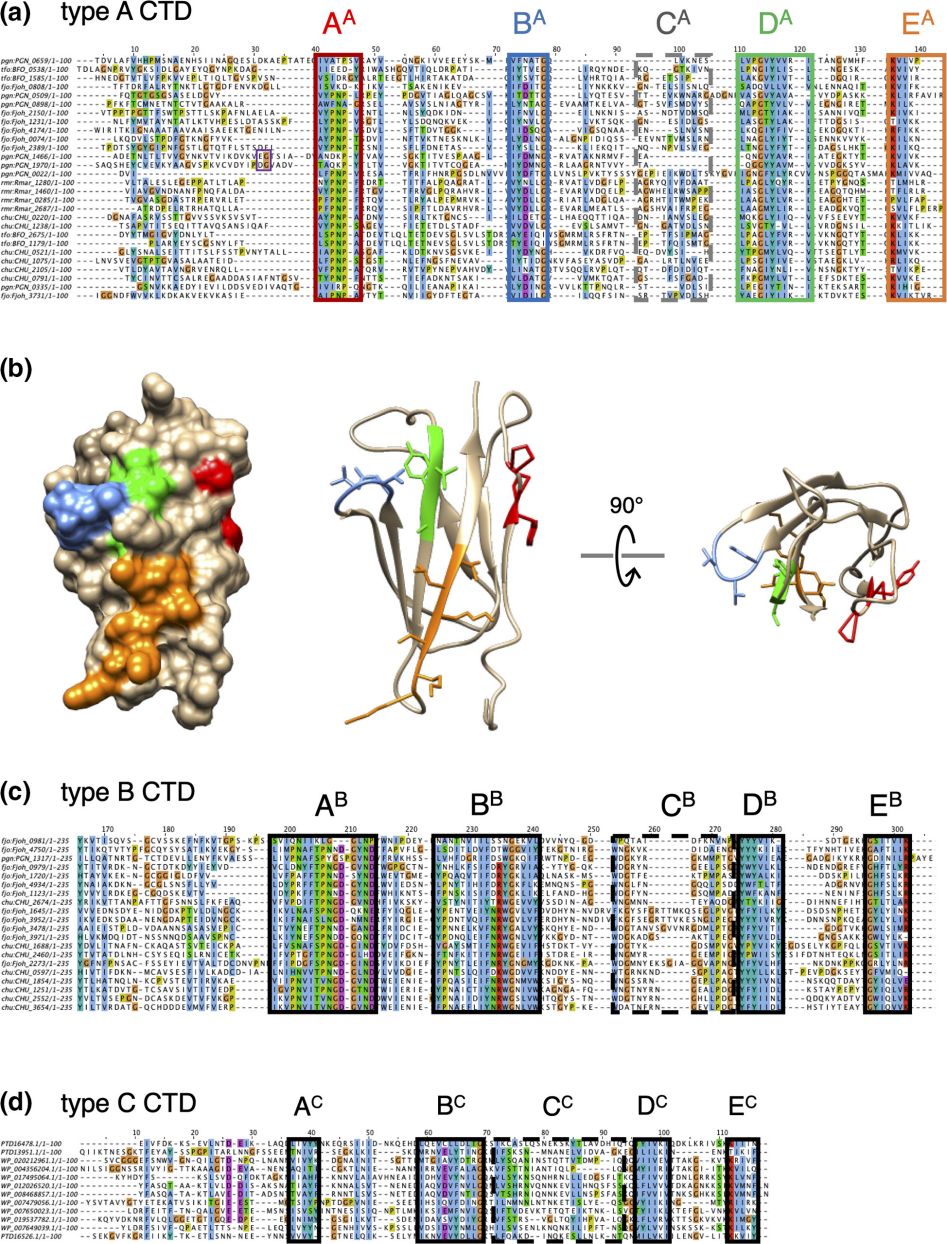
T9SS secretion signals. Sequence alignments of randomly selected type A (**a**), type B (**c**) and type C (**d**) CTDs from various *Bacteroidotoa* species using muscle [126] and edited using Jalview [127]. Residues with similar functional side-chains are shown in colour (blue, hydrophobic residue; orange, glycine; purple, negatively charged residue; red, positively charged residue; green, serine and threonine; yellow, proline). The different conserved motifs are framed. For type A CTDs, the residues upstream and downstream from the cleavage site, when experimentally validated, are framed in purple. (**b**) Surface representation (left) and ribbon representation (right) of the structure of the type A CTD from PorZ (PDB: 5M11 [50]). The A, B, D and E motifs are shown in colour [red, blue, green and orange, respectively; same colour as the frames in (a)] and with sticked side-chains in the ribbon representation.

The type A CTD (pfam family PF18962, TIGR04183) is the most represented T9SS sorting signal [46–48, 51, 52]. A sequence alignment of type A CTDs from diverse *Bacteroidota* revealed five motifs named A to E, from N- to C-terminal [46, 51]. While the A, B, D and E motifs are highly conserved, motif C is more degenerate [48, 51, 52] (Fig. 3a). The recent structures of the type A CTDs from the *P. gingivalis* RgpB, PorZ and Hbp35 substrates demonstrated a strict conservation of the fold, with a typical Ig-like organization comprising seven β-strands arranged as two anti-parallel β sheets [32, 53, 54]. In these structures, all the conserved residues of motifs A, B, D and E are exposed at the surface of the CTD and may therefore constitute binding interfaces to T9SS subunits (Fig. 3b). Mutagenesis of the CTD from the C-terminus revealed that the last 13 residues, and notably the conserved lysine residues of motif E, are required for efficient secretion [48, 52]. In addition, the last 22 amino acids of the HBP35 CTD, which encompass the D and E motifs, are sufficient to promote the heterologous secretion of GFP [47], questioning the importance of motifs A–C. One attractive explanation would be that motifs D and E are involved in crucial interactions with T9SS subunits, while other motifs are not required for secretion per se but participate in T9SS-CTD folding or facilitate substrate inspection at specific checkpoints along the type IX pathway. Indeed, motifs D and E were shown to regulate interactions with PorM and PorV, respectively [55], while an Alphafold model of the PorV/RgpB-CTD complex suggested that the B, D and E motifs mediate contacts with the extracellular loops of PorV [28].

### Type B CTD

Much less is known about the type B CTDs (pfam family PF13585, TIGR04131). Based on sequence homology searches, substrates with a type B CTD are less abundant than the effectors with a type A CTD in genomes. They can even be totally absent in some T9SS^+^ species [48, 49]. Transport of type B CTD substrates by the T9SS is still not understood but it is known that unlike the type A CTDs, their secretion does not require PorV, but specifically depends on a protein of the PorP/SprF family [49]. The genes encoding type B substrates and their cognate PorP/SprF proteins are usually clustered [49]. It is therefore proposed that PorP/SprF proteins are responsible for the anchoring of type B substrates to the cell surface [56]. In *P. gingivalis,* proper secretion of the unique type B substrate PG_1035 also requires PorE [56], an OM protein that binds the peptidoglycan [57, 58]. Similarly to type A CTDs, a sequence alignment of type B CTDs highlights five conserved motifs within the last 100 residues (Fig. 3c). Intriguingly, fusion of type B CTDs to GFP suggested that this region is not sufficient, but that a larger region of >200 residues is required for secretion in the medium [49]. A second significant difference with type A CTDs is that type B CTDs studied so far are probably not cleaved [48, 56].

### Type C CTD

The recently identified type C CTD family (protein domain family cl41395) includes the *Flavobacterium johnsoniae* ChiA chitinase and three putative T9SS substrates of *Flavobacterium columnare* [48, 50]. Kulkarni *et al.* demonstrated that the C-terminal 100-aa region of ChiA, which also comprises conserved motifs (Fig. 3d), is required for efficient secretion of GFP [48].

Although the identification of the T9SS-CTD as a sorting signal and the existence of different families of T9SS-CTDs with conserved motifs are now well established, many questions remain unanswered. How does the T9SS recognize different secretion signals? Does the secretion mechanism differ depending on the CTD family? Are these secretion signals linked to different functions or to different final localizations of the substrates?

## Post-translational modifications of T9SS effectors

During their journey along the T9SS pathway, and even after exiting the SprA/Sov translocon to reach the cell surface, substrates can be modified. These post-translational modifications may target the substrates to a specific location and/or regulate substrate activity.

### A sortase-like mechanism for T9SS-CTD cleavage and attachment

**Fig. 4.**
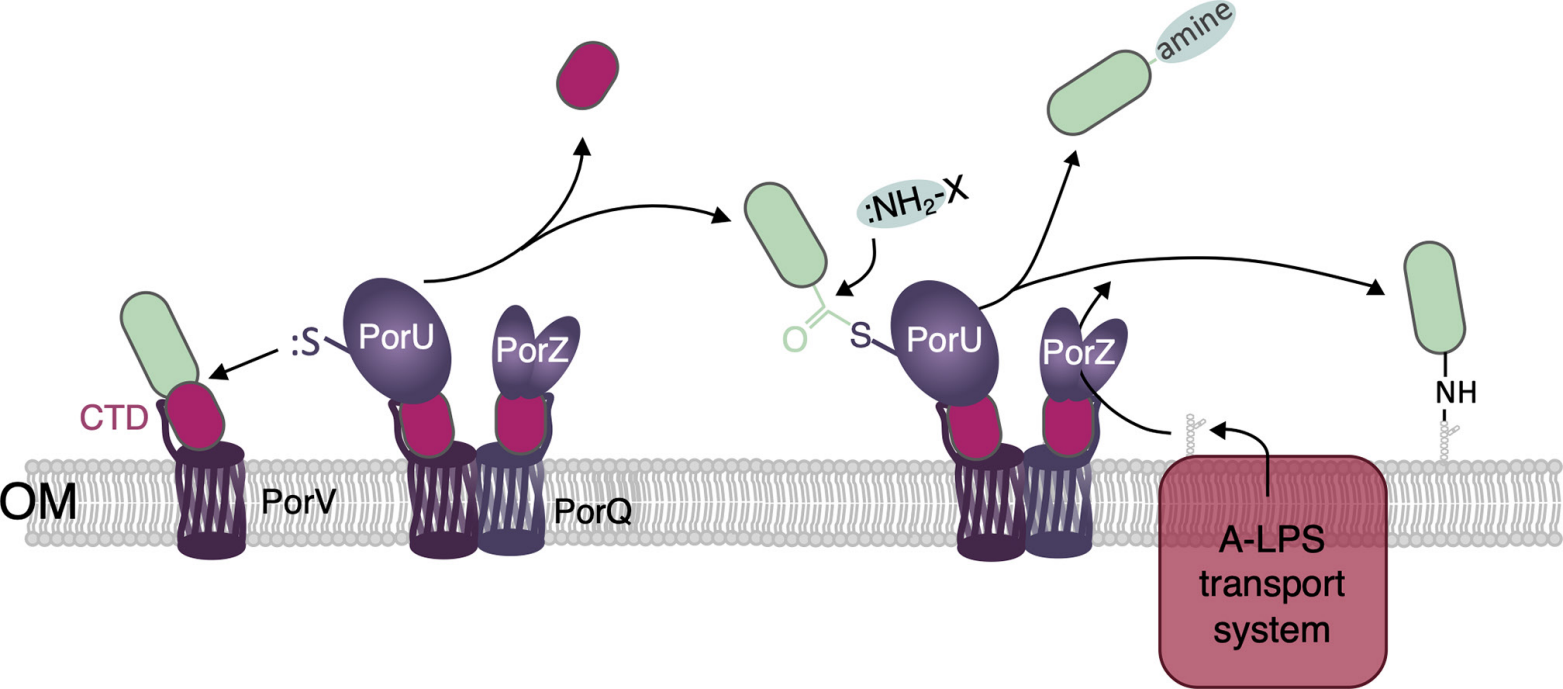
T9SS effector processing by the PorQUZ attachment complex. The T9SS effector is conveyed by the PorV shuttle to the PorQUZ complex. The PorU proteolytic domain cleaves the CTD, forming a thioester-bound complex between PorU and the processed substrate. The CTD is then either released in the medium or attached to the A-LPS after the nucleophilic attack of the free amino groups of primary amines, amino acids, short peptides or A-LPS. The A-LPS is synthesized by the Wba/Vim pathway and transported to PorU by the LPS transport machinery (pink) and PorZ.

A common feature of T9SS-dependent secretion is the anchoring of substrates to A-LPS at the cell surface. The current model proposes that after their translocation across the OM, substrates are shuttled by PorV to the PorQUZ attachment complex [27, 28] where they are processed by the multidomain PorU protein [31, 46] (Fig. 4). *P. gingivalis* PorU is a cysteine proteinase with a Cys/His catalytic dyad that cleaves the CTD at a T/E/D/N | G/A/S motif [31, 46] (Fig. 3a). This proteolysis is followed by a transpeptidation reaction that occurs between the last residue of the substrate processed form and the amine group of the 2-*N*-seryl, 3-*N*-acetylglucuronamide sugar of A-LPS synthesized by the Wbp/Vim pathway and provided by PorZ [31, 33, 34, 59] (Fig. 4). This process, reminiscent of the action of Gram-positive bacterial sortases that anchor proteins to the peptidoglycan, results in the attachment of T9SS substrates to the cell surface (Fig. 4). While the cleavage site has not been determined, PorU is also involved in removal of the N-terminal prodomain of gingipains in *P. gingivalis* [60].

Interestingly, PorU and PorZ are also CTD proteins and are thus T9SS substrates themselves; however, their CTDs have not been processed [30–33]. A recent study revealed that the PorU peptidase activity is inhibited upon dimerization [34]. Once a new PorU is shuttled to the attachment complex, a β-hairpin from one PorU monomer binds and inhibits the proteolytic domain of the second monomer [34]. Hence the PorU CTD is not removed and remains bound to PorV. The PorZ CTD domain is also retained to bind to the PorQ outer membrane β-barrel protein [27, 32, 35]. Although it is not yet known how the PorZ CTD remains uncleaved, one may hypothesize that specific handling of T9SS-CTD substrates by cognate PorV-like β-barrels protects the CTD from cleavage. This could be the case for PorZ/PorQ, as well as for type B substrates with PorP/SprF-like proteins.

It is noteworthy that not all T9SS substrates remained anchored to the cell surface, but a significant subset of them are freely released in the external milieu (Fig. 4). For these effectors, the CTD is removed by PorU and might be bound to amines or to the amine group of amino acids or short peptides present in the medium, instead of the amine group of A-LPS [31, 34] (Fig. 4). How PorU recognizes and selects effectors to be released or attached remains to be unravelled.

### *N*-Glycosylation of the CTD is required for secretion in *Cytophaga hutchinsonii*

*Cytophaga* species rely on the T9SS to secrete enzymes and other effectors at the cell surface that degrade cellulose and other polysaccharidic biopolymers [61–63]. The CTD of the Cel9A cellulase is *N*-glycosylated in the periplasm by the PglA-like CHU_3842 glycosyltransferase [64]. This modification, which occurs on the asparagine residue of a DXNXS motif, is necessary for Cel9A secretion and cell surface exposure [64, 65], suggesting that *N*-glycosylation may provide a means to regulate secretion of T9SS substrates. However, the physiological relevance of this modification and how it controls recognition and transport by the T9SS is not known.

### Phosphorylation of major *P. gingivalis* virulence factors may regulate their processing and secretion

Phosphorylation is a key post-translational modification regulating many processes, and type IX secretion is not an exception [66]. The phosphoproteome of *P. gingivalis* includes CTD-containing proteins such as gingipains, PPAD and RagB [66]. Substitution of the gingipain tyrosine residues that are phosphorylated strongly affected their secretion and processing [66], suggesting that phosphorylation of major *P. gingivalis* virulence factors may regulate their processing, activity and secretion. However, the kinase(s) responsible for the phosphorylation of these gingipains has not yet been identified.

## Biological functions of T9SS effectors

At this point in the review, the reader may draw the conclusion that any periplasmic-targeted protein fused to a T9SS-CTD signal could be secreted by the T9SS since the CTD is necessary and sufficient for secretion through the OM. Thus, the repertoire of T9SS effectors is tremendous, ranging from very small proteins to several hundred kilodalton adhesins such as SprB in *F. johnsoniae *, and the functions supported by the T9SS are very diverse. Indeed, the average number and the variety of putative T9SS effectors (CTD-containing proteins) per genome is higher than with any other secretion system, with no fewer than 230 potential effectors encoded in the genome of *Fluviicola taffensis* [48]. Hence, we will not list all the activities of T9SS effectors in this review, but will rather focus on major functions: virulence, motility and polysaccharide utilization (Table 1).

**Table 1.** T9SS effector proteins

T9SS effector type	Activity or biological role	Examples and/or bacterial species	Reference(s)
**Proteases**			
Gingipains	Degradation of proteins from host tissue, processing of fimbriae subunits	* P. gingivalis* RgpB, Kgp	[68, 69, 71–76]
Subtilisin (serine protease)	Host complement evasion, and degradation of gelatin, fibrinogen, and LL-37	* Riemerella anatipestifer* SspA	[128]
Peptidase	Host tissue degradation	* F. columnare *	[50]
**Peptidylarginine deiminase**	Citrullination of host proteins	* P. gingivalis* PPAD	[81, 82]
**Metallophosphoesterase**	Phosphatase involved in virulence	* R. anatipestifer *	[129]
**Endonuclease**	Broad-range nuclease involved in virulence	* R. anatipestifer *	[130]
**CAZymes**			
Chitinase	Chitin degradation	* F. johnsoniae* ChiA	[9]
Cellulase	Cellulose degradation	* Cytophaga hutchinsonii* Cel9A	[61, 63]
Xylanase	Xylan degradation	* Roseithermus sacchariphilus *	[131]
**Haem-binding protein**	Haem uptake and utilization	* P. gingivalis* Hbp35	[120]
**S-layer protein**	Cell-surface protective layer	* Tannerella forsythia* TfsA and TfsB	[10, 83]
**Adhesins**	Gliding motility	* F. johnsoniae* SprB and RemA	[8, 88, 89]

### Virulence

Since *P. gingivalis* was shown to be a critical periodontal pathogen, research focusing on the mechanism of its virulence has received extensive attention. In 2010, the groups of McBride and Nakayama discovered the T9SS, which was initially called ‘Por secretion system’ (PorSS) [7]. Por refers to the porphyrin black pigment that accumulates at the surface of wild-type *P. gingivalis* colonies grown on blood agar [67]. This phenomenon relies on the activity of specific surface-anchored proteases called gingipains [68]. Mutations generating apigmented colonies mapped to genes responsible for gingipain secretion, cell surface exposition or maturation. Gingipains are Cys/His proteases that cleave at arginine (Rgp) or lysine (Kgp) residues [69, 70]. The gingipains are exposed at the cell surface and assemble an electron-dense layer at the surface of the bacterium [71]. The gingipains target host proteins such as matrix proteins (collagen, tight-junction proteins, etc.) [72, 73] and thus participate in the degradation of the periodontal tissues, allowing penetration of the bacterium across the epithelial barrier. Gingipains are also indirectly involved in adhesion to host cells by processing the pilin subunits of Mfa fimbriae and Type V pili [74–76]. In addition to gingipains, *P. gingivalis* uses its T9SS to secrete toxins that promote binding to host epithelial gingival cells, citrullination of proteins, and inhibition of the host immune response [76–80]. The T9SS-secreted protein PPAD (* Porphyromonas* peptidyl arginine deiminase) converts arginine to citrulline and induces aggregation and/or unfolding of target proteins by increasing their overall hydrophobicity [81]. PPAD is thus an important virulence factor responsible for the development of periodontitis and rheumatoid arthritis [78, 82]. Here, we will not expand further on the predominant role of T9SS effectors in the virulence of *P. gingivalis *, which has been covered recently [1, 5, 80]. Other *Bacteroidota* pathogens secrete their virulence factors *via* their T9SS, such as the oral human pathogen *Tannerella forsythia *, the fish pathogens *Flavobacterium columnare* and *F. psychrophylum*, and the duck pathogen *Riemerella anatipestifer* [10, 46, 50, 83–86].

### Motility

T9SS research is also intimately linked to gliding motility. Many *Bacteroidota* members crawl over solid surfaces using cell surface adhesins [42, 87]. On motility plates, *F. johnsoniae* colonies expand until reaching the limits of the Petri dish. Elegant genetic screens based on the isolation of non-motile mutants led to the identification of the major adhesin SprB [88], the minor adhesin RemA [89], components of the T9SS [7, 23, 37, 90] and additional proteins grouped into gliding-specific modules [22, 36, 38–41, 91–93]. In *F. johnsoniae *, the major adhesin SprB moves rapidly at the cell surface, describing a closed helical loop from pole to pole [8, 11, 94]. Once attached to the substratum, the collective force generated by movement of SprB molecules is thought to act as a traction force on the substratum. As a result, the cell body is displaced in a screw-like rotational movement opposite to the direction of SprB bound to the substratum [8, 95]. Although this is reminiscent of the focal adhesion mechanism used by *Myxococcus xanthus* to glide [96], how SprB moves rapidly in a spiral motion on the cell surface remains mysterious. However, it is clear that this process is intimately entangled with type IX-dependent secretion.

The T9SS was shown to be responsible for the secretion of the motility adhesins, and hence inactivation of the T9SS prevents gliding motility [7, 88, 89, 97]. Interestingly, SprB possesses a type B CTD and requires SprF whereas RemA possesses a type A CTD and requires PorV for efficient secretion. How both types of effectors are targeted to the gliding machinery is not known. However, the situation is even more complicated. The activity of the T9SS molecular motor, GldLM, is not only required for SprB secretion but also for the movement of SprB at the cell surface [14, 19], suggesting that this proton-dependent motor also powers SprB dynamics [98, 99]. Shrivastava and Berg proposed a rack and pinion model to explain how the force provided by GldLM triggers SprB movement [100, 101]. In their model, the GldLM torque is transmitted to a ‘gearbox’ made of pinions that move a tread carrying the adhesins. Furthermore, a fixed helical track may guide this tread. The nature of the tread is still unknown but recent data suggest that GldJ may participate in the formation of this structure. First, the extreme C-terminal region of GldJ (8–13 aa) was dispensable for secretion via the T9SS but was required for SprB dynamics [24], suggesting a link between GldJ and the gliding apparatus. Indeed, GldJ is required for and present in filamentous structures underneath the OM that could serve as rails to guide a moving tread [102].

### Biopolymer degradation and utilization

Many *Bacteroidota* from *Flavobacterium *, *Cytophaga* and *Cellulophaga* and many other genera are environmental bacteria from diverse ecological niches [12, 103]. They use their T9SSs to secrete biopolymer-degrading enzymes, such as cellulases that degrade cellulose, the main component of the plant cell wall, agarases that degrade algal agarose and chitinases that degrade chitin, a structural component of molluscs, insects, crustaceans and fungi [9, 62, 63, 104–106]. The secretion of polysaccharide-degradation enzymes is not restricted to environmental strains, as many gut commensal *Bacteroidota* also secrete such enzymes via the T9SS [105, 107]. These activities provide nutrients and allow bacteria to establish themselves in their niches [108, 109].

### S-layer formation

The S-layer is an additional structure surrounding some bacteria and archaea [110–112]. In Gram-negative bacteria, the S-layer is made of glycoproteins attached to the LPS and confers protection and resistance to mechanical stress and external aggressions [110, 113, 114]. In *Tannerella forsythia *, the T9SS secretes two glycoproteins, TfsA and TfsB, that self-assemble to form a two-dimensional crystalline S-layer lattice that covers then entire cell [10, 83]. TfsA and TfsB are *O*-glycosylated by addition of a branched dekasaccharide [115]. In T9SS mutants, TfsA and TfsB remain sequestered in the periplasm but are *O-*glycosylated, demonstrating that post-translational modifications and secretion are uncoupled [10]. By being involved in the adhesion to and invasion of gingival epithelial cells and in biofilm formation on mucin, the TfsAB S-layer plays a key role in *T. forsythia* virulence [116–118].

### Haem acquisition

Many bacterial species require haem as a source of iron for their survival and, in some cases, to establish an infection. The *P. gingivalis* T9SS deploys the Hbp35 haem-binding protein to capture haem from the environment or from host haem-containing proteins [47, 119, 120]. In addition to *P. gingivalis* gingipains, proteases required for the degradation of haem proteins are also secreted through the T9SS in *Prevotella intermedia* and *T. forsythia* [121, 122].

### Is there much to discover about T9SS effector functions?

Because most T9SS research has focused on a few model bacteria, only a tiny fraction of the huge T9SS effector repertoire has been identified and characterized. This leaves many possible areas to prospect, and notably the emerging role of the T9SS in the interplay between *Bacteroidota* species and their hosts. For example, recent evidence suggest that the T9SS is involved in symbiotic or pathogenic relationships of marine *Bacteroidota* with microalgae, such as for the interaction of *Dyadobacter* with *Micrasterias radians* [123–125].

While the field has made significant advances in recent years, many aspects of the T9SS remain to be clarified. In addition to a better understanding of the composition and architecture of the secretion apparatus itself, the translocation pathway of effectors from the periplasm to the cell exterior needs to be deciphered. How are these effectors recognized and selected, and what are the hierarchical contacts during their transport through the T9SS? It is still unclear at which stage of the translocation pathway the effectors acquire their definitive folding. Many questions also remain on how the effectors are post-translationally modified, and to what extent these modifications impact their secretion and activities. Finally, how the additional modules are connected to the T9SS and how they provide new functions are important questions to address. Using multidisciplinary approaches and novel technologies, future efforts will probably provide valuable insights and will pave the way to elucidating the mechanism of transport and the versatility of effector activities.
